# Intravenous Ringers lactate versus normal saline for predominantly mild acute pancreatitis in a Nepalese Tertiary Hospital

**DOI:** 10.1371/journal.pone.0263221

**Published:** 2022-01-28

**Authors:** Binod Karki, Suresh Thapa, Dibas Khadka, Sanjit Karki, Roshan Shrestha, Ajit Khanal, Ramila Shrestha, Bidhan Nidhi Paudel

**Affiliations:** 1 Department of Medicine, Nepalese Army Institute of Health Sciences, Kathmandu, Nepal; 2 Department of Medicine, Pokhara Academy of Health Sciences, Pokhara, Nepal; 3 Gastroenterology Unit, Department of Medicine, National Academy of Medical Sciences, Kathmandu, Nepal; 4 Department of Medicine, Patan Academy of Health Sciences, Lalitpur, Nepal; University of Cambridge, UNITED KINGDOM

## Abstract

**Background:**

Acute pancreatitis (AP) is a common presentation in patients admitted with acute abdomen. Whether Ringers lactate (RL) or Normal Saline (NS) as a resuscitation fluid is better still remains unclear. The aim of this study is to compare the efficacy of RL and NS in terms of control of systemic inflammation by measuring indirect markers specifically Systemic Inflammation Response Syndrome (SIRS) scores and C- Reactive Protein (CRP) level.

**Methods:**

This was an open label randomized trial conducted in a tertiary level hospital of Nepal. Ethical approval was obtained prior to the study. Patients with acute pancreatitis were randomized to either RL or NS group for the fluid resuscitation. The fluid was given as per the study protocol for three days for hydration. Baseline SIRS and CRP were recorded on admission and subsequently as defined. All the data were analyzed using SPSS ver 20.0 software.

**Results:**

Total 51 patients were enrolled, 26 in RL and 25 in NS group. The commonest etiology of AP was alcohol (84.31%). SIRS was present in 46.2% and 64.0% of patients in RL and NS group respectively (p = 0.20) on admission. At least one SIRS criteria was still present in 44.0% of patients in the NS group compared to only 15.4% in the RL group after 24 hours (p = 0.025). The baseline CRP were comparable in both the groups. However after 72 hours, the increment of CRP was more in the NS group compared to the RL group; median value of 14.2 mg/dl (12.15, 16.45) and 22.2 mg/dl (18.20, 30.60) in RL and NS group respectively (p<0.001).

**Conclusions:**

Ringers lactate was associated with a reduction in systemic inflammation compared to normal saline in patients with acute pancreatitis. Incidence of SIRS at 72 hours and occurrence of local complications were however similar in both the groups.

## Introduction

Acute pancreatitis is often associated with a systemic inflammatory response [1]. The inflammation can either resolve spontaneously or can progress to local complication leading to necrosis of the pancreas itself or the surrounding tissues. Early aggressive fluid resuscitation is key in initial management of acute pancreatitis. Fluid resuscitation is believed to play an important role in the prevention of complications such as pancreatic necrosis and organ failure by preserving pancreatic micro circulation [2, 3].

Crystalloids volume resuscitation with both Normal Saline (NS) and Ringer’s Lactate (RL) is preferred for the early phase of acute pancreatitis. However, infusion of large volume of NS can lead to development of a hyperchloremic metabolic acidosis and the choice of initial fluid is still unclear [4, 5]. Ringers lactate is an attractive alternative due to its pH balance. However choice of fluids among different crystalloids so far is based upon expert opinion only. The severity of acute pancreatitis can be measured with various markers and inflammation scores such as C- Reactive Protein (CRP) titer and Systemic Inflammation Response Syndrome (SIRS) parameters [6, 7].

### Aim

The aim of the study is to compare the reduction of the SIRS scores and the CRP titer in acute pancreatitis during initial resuscitation with RL compared to NS.

## Methods

This was an open label randomized study done in Bir Hospital of Nepal. Bir Hospital is 535-bed tertiary level referral government hospital located at Kathmandu, which is affiliated with National Academy of Medical Sciences (NAMS). The study was conducted from October 2018 to June 2019. The trial was registered in University hospital Medical Information Network (UMIN) database as UMIN000035295. The trial was registered after enrollment of patient started as there was no national trial registry of Nepal at the time of commencement of research. The authors confirm that all ongoing and related trials for this intervention are registered. Approval was obtained from the Institutional Review Board (IRB) of National Academy of Medical Sciences, Bir Hospital, Kathmandu, (Approval reference number 709).

### Inclusion criteria

A written consent was obtained from all the participants. Patients aged 18 years or older who were admitted with a diagnosis of acute pancreatitis were eligible for study participation. Diagnosis was established by the presence of two or more of the following criteria: (1) epigastric abdominal pain, (2) elevation in serum amylase and/or lipase level greater than 3 times the upper limit of normal, (3) confirmatory findings on cross-sectional imaging. Acute pancreatitis was graded as mild, moderately severe and severe. Mild acute pancreatitis was defined as—No organ failure, or absence of local or systemic complications. Moderately severe acute pancreatitis was defined as organ failure that resolves within 48 hours (transient organ failure) associated with local or systemic complications without persistent organ failure. Severe acute pancreatitis was defined as persistent organ failure (>48 h) associated with either single or multiple organ failure.

### Exclusion criteria

Patients were excluded from participation if they met any of the following criteria: Age below 18 years, presenting to the hospital for more than 48 hours of symptoms, referred after initial resuscitation in another hospital, and known history of severe cardiovascular, respiratory, renal, hepatic, hematologic, or immunologic disease defined as (1) greater than New York Heart Association class II heart failure, (2) active myocardial ischemia or (3) cardiovascular intervention within previous 60 days, (4) history of cirrhosis or (5) chronic kidney disease with creatinine clearance < 40 mL/min, or (6) chronic obstructive pulmonary disease with requirement for home oxygen.

The primary outcome of the study was to measure the difference in CRP level and SIRS score at the admission and subsequently. SIRS score were recorded as present or not present and if present as score 1–4 for the following parameters: Temperature<36°C (96.8°F) or >38°C (100.4°F); Heart rate>90/min; Respiratory rate>20/min; WBC(<4000/mm^3^) or (>12,000/mm^3^) or 10% bands. SIRS was diagnosed if at least two scores were fulfilled. CRP measurement was done by turbidometry method and expressed in quantitative CRP as mg/dl.

SIRS was recorded at 24 hours and 72 hours of presentation. CRP was reassessed at the end of 72 hours. The secondary outcomes were to measure the difference in the occurrence of local complications, length of hospital stay and in hospital mortality.

For sample size estimation we used CRP level as the change in the systemic inflammatory status. In a prospective cohort study by Acevedo-Piedra NG et al. [8], patients who received fluid resuscitation based on NS, mean blood CRP levels was 160 mg/l (standard deviation 111). We aimed to detect a 100-mg/l difference with at least 80% power, at a two-sided alpha level of 5%. It was calculated that 25 patients in each arm would be needed to have 95% confidence interval.

### Intervention details

All the patients diagnosed as acute pancreatitis using the Atlanta classification [9] were randomized to fluid resuscitation using either RL or NS. The method of randomization was using a sealed envelop technique. The fluid, to which each patient was randomized was administered initially as a bolus of 10ml/kg in 60 minutes followed by infusion at the rate of 1.5 ml/kg/hour till they could be started on an oral diet. All patients received additional one liter of 5% dextrose per 24 hours. Patients who tolerated a diet orally were given intravenous fluids as per daily was decided by the treating clinician. Additional potassium chloride was supplemented as per daily serum biochemistry result. Adequate fluid replacement were assessed by an improvement in vital signs (goal heart rate <120 beats/minute, mean arterial pressure between 65 to 85 mm Hg), urine output (>0.5 to 1 ml/kg/hour) and BUN over 24 hours, particularly if they were high at the onset. All patients were followed till the time of hospital discharge or death. The patients who developed either peri-pancreatic fluid collection or pancreatic necrosis were followed up to four weeks. The hospital safety board had the authority to terminate the study if any concern of patient safety due to the research protocol occurred.

Data were analyzed on an intention-to-treat basis. Continuous data were analyzed using mean and standard deviation (SD) or median depending on the variable distribution. Differences between the two groups with continuous data were assessed using student-t test for normal and Mann-Whitney U test for non-normal distributions.

Quantitative data were described using percentages and compared by the chi-square A two-sided p value of less than 0.05 was considered statistically significant. All statistical calculations were performed with SPSS ver 20.0.

## Results

**Fig 1:**
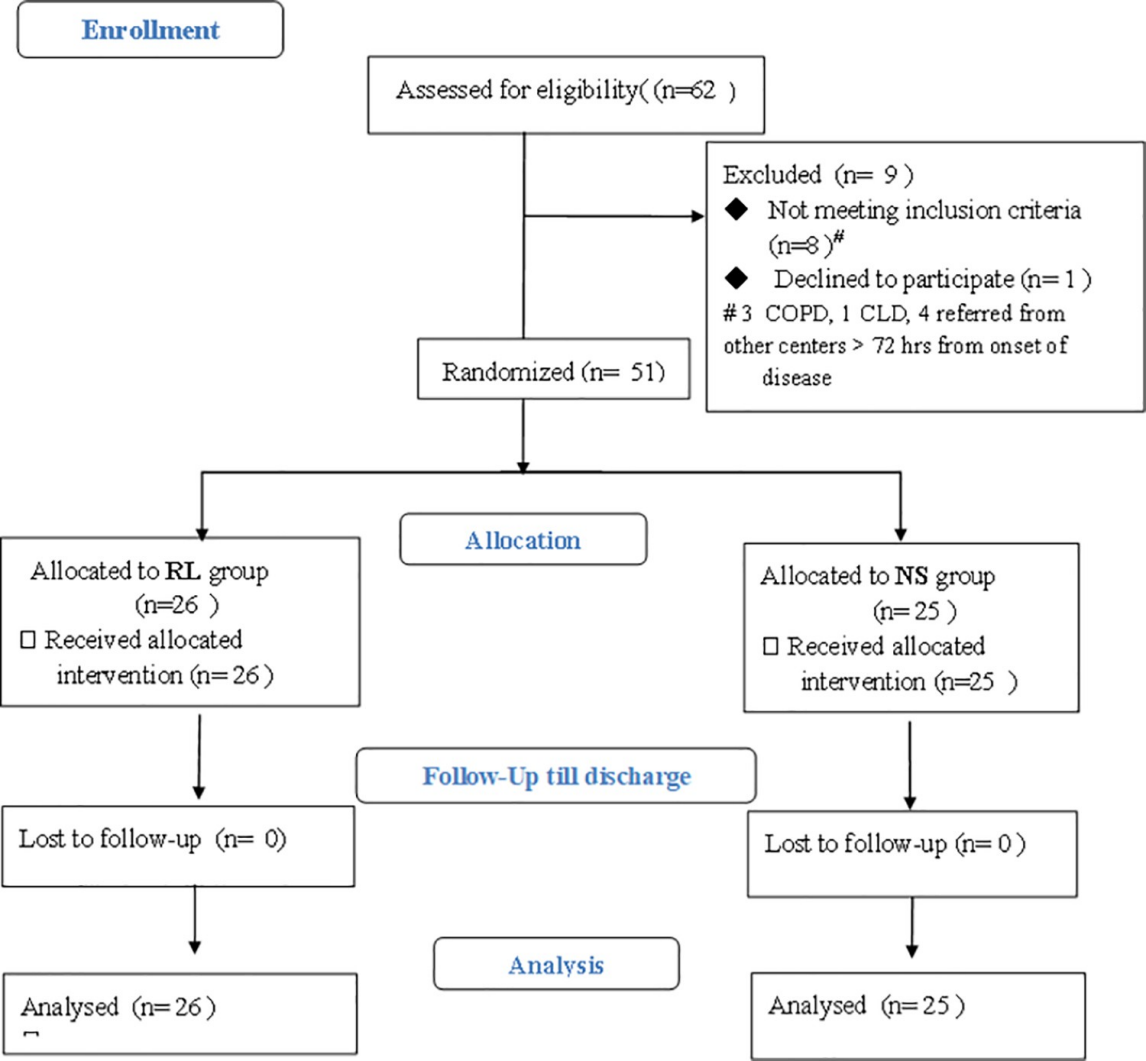
Consort flow diagram of the participants in the trial.

A total of 62 patients were assessed for the eligibility during the total study period. Nine patients were excluded; eight had various co -morbid conditions including COPD, renal failure or heart failure (Fig 1). These conditions were excluded as these patients could not be managed with aggressive fluid therapy. One patient declined the consent. A total of 51 patients were randomized, 26 patients in the RL group and 25 patients in the NS group (flow chart). Out of total 51 patients, the mean age was 41.33 ± 14.17 years. There were 46.2% and 52.0% of total patients in the age group of 21–40 years in RL and NS group respectively. The most common etiology of the acute pancreatitis in the study was alcohol consumption (84.3%) followed by gall stone diseases (7.8%). The rest of the cases were idiopathic. Most patients had mild acute pancreatitis. The baseline characteristics of the study population were comparable in both the groups except for white blood cell counts which was higher in the NS group compared to RL although the mean in both the groups was more than the cut off value required for the definition of SIRS (Table 1).

**Table 1:** Baseline characteristics of the study population.

Variables	RL (N = 26)	NS (N = 25)	P value
Gender (Male), n (%)	25 (96.2%)	1 (3.8%)	0.024
Age group: 20 yrs or less	1 (3.8%)	1 (4.0%)	
21–40 yrs	12 (46.2%)	13 (52.0%)	
41–60 yrs	11 (42.3%)	8 (32.0%)	
More than 60 yrs	2 (7.7%)	3 (12.0%)	
Hypertension, n (%)	3 (11.5%)	2 (8.6%)	0.67
Diabetes, n (%)	2 (7.7%)	3 (12.0%)	0.61
Pulse (Mean, bpm ±SD)	88.04± 8.12	90.92± 13.46	0.35
SBP (Mean, mm Hg±SD)	116.54 ±13.54	118.80 ±25.38	0.69
DBP (Mean, mm Hg±SD)	75.85 ±8.52	74.88± 11.56	0.73
WBC (Mean, per cmm±SD)	11051± 3503	15116± 7039	0.01
Urea (Mean, mg/dl±SD)	36.23 ±11.6	51.08 ±39.41	0.07
Creatinine (Mean, mg/dl±SD)	0.95 ± 0.19	1.2 ±0.67	0.03
Amylase (Median, mg/dl, IQR)	1024 (532,1475)	1113 (362,2072)	0.89
BISAP score, Median (IQR)	1(0,1)	1(1,2)	0.12
SIRS present on admission, n (%)	12/26 (46.2)	14/25 (64.0%)	0.20
CRP baseline (mg/dl) Median (IQR)	4.6 (3.18,5.2)	4.2 (3.22,4.95)	0.47

**Fig 2:**
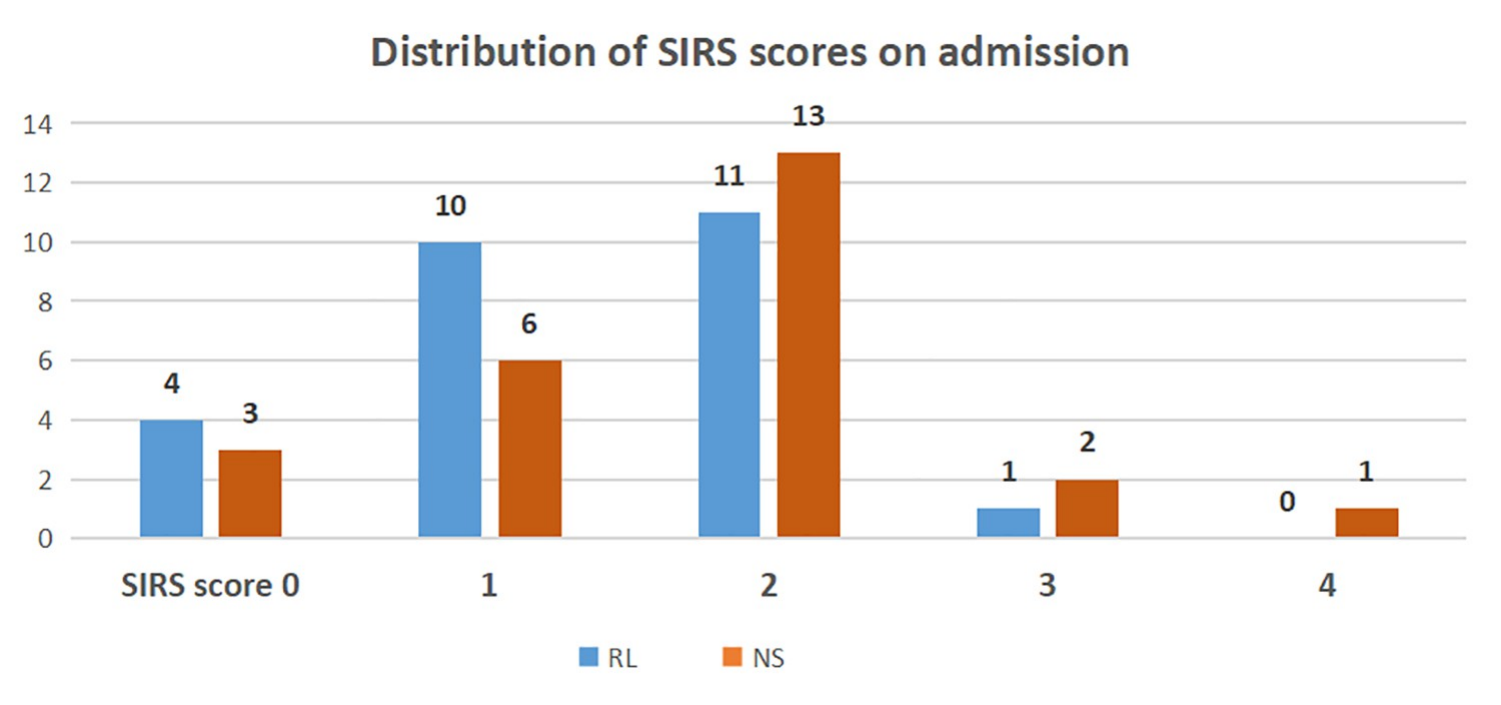
Distribution of SIRS score in the study population. RL: Ringers lactate, NS: Normal Saline.

There were 46.2% and 64.0% patients with SIRS diagnostic criteria at the time of randomization in RL and NS group respectively (Fig 2). When the number of SIRS parameters were evaluated for the individual patient, most of the patients in both the groups had at least two elevated parameters. One patient (4.0%) in NS group only had all four scores during admission (Fig 2).

**Fig 3:**
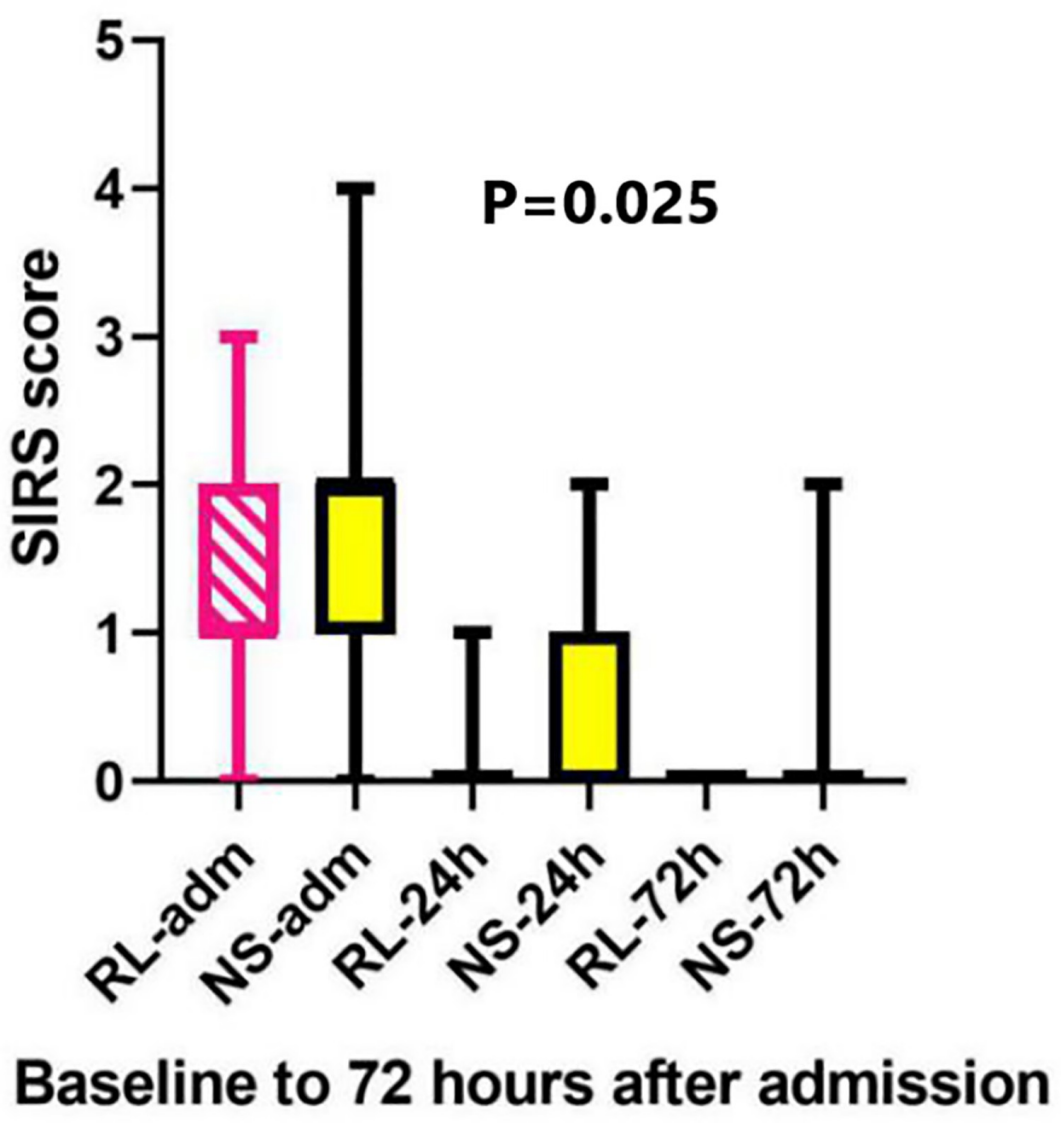
Box plot presentation of SIRS score in two groups. RL-adm: Ringer Lactate group at admission, RL-24 h: Ringer Lactate group at 24 hours, RL-72h: Ringer Lactate group at 72 hours, NS-adm: Normal Saline group at admission, NS-24h: Normal Saline group at 24 hours, NS-72h: Normal saline group at 72 hours.

After 24 hours of randomization, two patients in NS group had SIRS compared to none in RL group. Four (15.4%) patients in RL group had at least one score present at 24 hours compared to 11(44%) of patients in NS group (p = 0.025). After 72 hours, three patients in NS group still had at least one SIRS score compared to none in RL group with SIRS diagnosis even after 72 hours of admission The median SIRS on admission and on subsequent days have been illustrated in the box plot (Fig 3).

**Fig 4:**
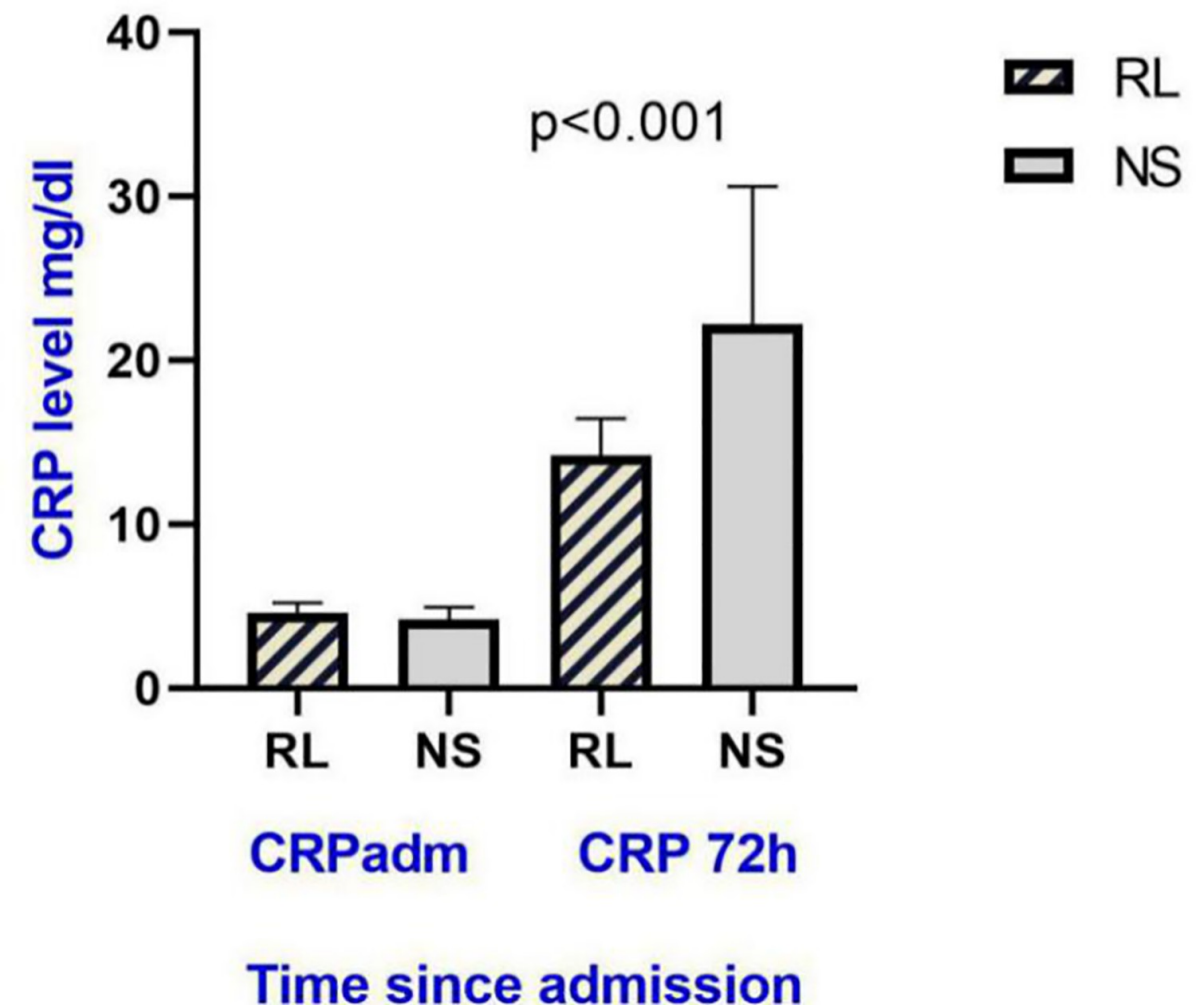
Bar diagram showing the CRP titre and its increment at 72 hours from the baseline values in the study population. CRP adm: CRP level at admission, CRP 72h: CRP lever after 72 hours of admission, RL: Ringers lactate, NS: Normal Saline.

The median CRP at the baseline in RL and NS groups were 4.6 mg/dl (3.18, 5.2) and 4.2 mg/dl (3.22, 4.95) respectively. At 72 hours, the increment in CRP was higher in the NS group compared with the RL group. The median value of CRP were 14.2 in RL vs 22.2 in NS respectively and the difference was statistically significant (p <0.001). The percentage increment of CRP from baseline to the value recorded at 72 hours was also significantly different between the two groups (Fig 4).

The incidence of local complications, length of hospital stay and the mortality was not significantly different in both the study groups (Table 2). The local complications were further categorized into peri-pancreatic fluid collection, acute pancreatic necrosis, pseudo cyst and walled off necrosis as per the defining criteria from modified Atlanta classification.^9^ None of the patients in the study require hemodialysis support. All three patients with walled off necrosis were referred to other tertiary care centers for endoscopic therapy procedures and were lost to follow up. One patient in NS group developed severe acute respiratory distress syndrome and had to be kept under mechanical ventilator and subsequently died. There was also a non-significant trend towards more frequent moderately severe/severe pancreatitis with NS, 42.30% (11/25) versus RL, 23.07% (6/26) [p = 0.11].

**Table 2:** Comparison of secondary outcomes in the study populations.

Complications (local)	Ringer’s Lactate	Normal Saline	p value
	N = 26	N = 25	
Over all local complication, n (%)	6 (23.1%)	7/25 (28%)	0.68
Details of local complications			
Peri-pancreatic fluid collection, n (%)	4 (15.38%)	4 (16%)	
Acute necrotic collection, n (%)	2 (7.69%)	3 (12%)	
Pseudocyst, n (%)	1 (3.84%)	1 (4%)	
Walled off necrosis, n (%)	1 (3.84%)	2 (8%)	
Hospital stay in days (mean) ±SD	5.15 ± 0.09	6.20 ± 2.5	0.06
In hospital mortality, n (%)	0 (0%)	1 (4%)	0.90

## Discussion

The mean age of our study population was similar to other Nepalese study [10] where the mean age was 42.7±16.5. In other studies done outside Nepal in which intravenous normal saline and Ringers lactate were compared, the median age was 51 and 51.2 years respectively [11, 12]. The age distribution in our study population showed that majority were in the age group of 20–60 years indicating acute pancreatitis being the disease of young.

The most common etiology of acute pancreatitis in our study was alcohol which is different from other studies where it was predominantly gall stone pancreatitis [13, 14]. In our hospital, patients with acute pancreatitis could be admitted into either a medical or surgical ward. Thus some of the patients can be lost to capture. Secondly, we did not include patients of biliary pancreatitis who underwent Endoscopic Retrograde Cholangiography. The pathogenesis of alcohol related pancreatitis is different from that of gall stone pancreatitis. The expected response to intravenous fluid administration is likely to be different. In addition, the results may not be generalizable to a cohort of patients with AP in which higher proportion of cases were biliary in origin.

The prevalence of SIRS criteria ≥2 was higher in the NS group. The baseline SIRS scores were not significantly different between the two groups. However at the end of 24 hours, fewer patients in the RL group had at least one SIRS criteria compared to the NS group. This suggests that the RL may be better in achieving the control over the systemic inflammation.

The SIRS lowering effect of RL over NS was also seen in the study by Wu B et al. [11], where participants who received RL had a significant reduction in SIRS at 24 hours from baseline compared with those who received NS for initial resuscitation (84% reduction in RL vs 0% in NS, p = .035). Choosakul S et al. [14] who followed the similar methodology in fluid resuscitation and demonstrated that at 24 hours, the prevalence of SIRS was 2 (8.7%) vs 9 (37.5%) in RL and NS group respectively (p = 0.02). In a study by De-Madaria et al. [15], at 24 hours of randomization, the change is SIRS was not significantly different between the two groups (p = 0.87). However in their study, they conducted in vitro experiment, where Lactated ringer inhibited the induction of inflammatory phenotype of macrophages and NF-kB activation. This suggests the anti inflammatory effect of Ringers lactate. Recently, Lee A et al. [16] showed in a similar randomized control trial that the need of intensive care was lesser and the length of hospital stay was shorter in the Lactated ringer’s group compared to the normal saline group although there was no difference in the prevalence of SIRS at 24 hours from the admission.

The CRP level at baseline and at 72 hours in our study population was also significantly different. At the end of 72 hours, it was seen that the increment of CRP occurred in both the groups. However, the increment was comparatively higher in the NS group. Most of the other studies also showed similar results in terms of CRP titre. Wu B et al. [11] showed that after 24 hours, subjects who were randomized to RL had lower CRP levels compared with participants in the NS treatment arms (mean CRP, 51 mg/L RL vs 104 mg/L NS; ANOVA, p = 0.018). De-Madaria et al. [15] also showed lower CRP titre at 48 hours and 72 hours of admission in the RL group compared to the NS group. Choosakul S et al. [14] showed the median CRP change at 48 hours were +18.19 (4.43,7.83) and +31.73 (1.97,27.2) respectively for RL and NS group though not statistically significant (p = 0.756).

The beneficial effects of RL in terms of alleviation of systemic inflammation can be explained by mainly two mechanisms; an indirect effect related to NS-associated metabolic acidosis and a direct anti-inflammatory effect of RL. Acidosis has been associated with inflammation and necrosis in experimental models. The evidence of hyperchloremic metabolic acidosis induced by normal saline infusion have been showed in a study by Scheingraber S et al. [5]. The evidence of metabolic acidosis induced by NS was also shown by the study carried out in a group of health volunteers where serum bicarbonate concentration was significantly lower after saline than after Hartmann’s (P = 0.008) [17].

The deleterious effect of NS over RL has been shown in other conditions besides acute pancreatitis where statistically significant decrease in pH, excess of base and a significant increase in serum chloride occurred in patients receiving saline during surgery [18–20]. The harmful effect of acidic medium has been explained by NF-kB activation and release of pro inflammatory cytokines as well as acidosis associated pathologies such as post ischemic inflammatory process [21, 22].

Our studies have several limitations. This was an open label randomized trial and not a blinded one. This was a single centre study and we could not collect the long term data in terms of mortality beyond the initial hospital stay.

## Conclusions

Ringer’s lactate was superior to normal saline in reduction of SIRS especially in first 24 hours. There was also significant reduction in CRP and SIRS parameters after 72 hours in RL group. Incidence of local complications and in hospital outcomes were however similar in both the groups.

## Supporting information

S1 Checklist CONSORT 2010 checklist for article acute pancreatitis. (DOCX)

S1 File IRB clearance pancreatitis research. (PDF)

S2 File Pancreatitis article proposal. (PDF)
