# Supplementary material for: Intravenous Ringers lactate versus normal saline for predominantly mild acute pancreatitis in a Nepalese Tertiary Hospital
Source: PLoS One. 2022 Jan 28;17(1):e0263221. doi: 10.1371/journal.pone.0263221 (PMC9126573; doi:10.1371/journal.pone.0263221)
Supplement: S1 File — (PDF) [file pone.0263221.s002.pdf]

National Academy of Medical Sciences
Bir Hospital
Mahaboudha, Kathmandu
Nepal

NATIONAL ACADEMY OF MEDICAL SCIENCES
BIR HOSPITAL, NEPAL

Ref. No. 709

Phone No.: 4230710
Fax No.: 4247032

Date: 2075/6/15
October 1, 2018

Approval of Thesis proposal

Name of the candidate/ researcher: BINOD KARKI
Registration No.: GASTRO -8
Program and year: DM GASTROENTEROLOGY , DECEMBER 2016.
Department: GASTROENTEROLOGY

Title of the Proposal: "Choice of Initial Resuscitating Fluids In The Outcome Of Acute Pancreatitis"
Name of Guide: PROF. RAMILA SHRESTHA
Name of Co-guide: - None
Study design: RANDOMIZED CONTROLLED TRIAL
Sample size: 38

General Objective:
To evaluate the effect of initial resuscitation with Ringers Lactate versus Normal Saline on the outcome of acute pancreatitis
Specific Objectives:
1. To analyze the difference in CRP in two groups as marker of systemic inflammation.
2. To analyze the difference in SIRS in two groups as marker of systemic inflammation.
3. To analyze the incidence of severity of diseases in both the groups as per the revised Atlanta classification.
4. To analyze the difference in complications of acute pancreatitis in both the groups.
5. To analyze the difference in length of hospital stay in both the groups

Ethical clearance: Ethical clearance given

Date of IRB approval: 2075/6/15 (October 1, 2018)

The following thesis proposal has been accepted by Institutional Review Board (IRB) of National Academy of Medical Sciences, Bir Hospital, Kathmandu.

You are advised to conduct the study according to the technical and ethical standards and rules and regulation laid down by concerned institute /NAMS/ IRC and required to submit a copy of your final report/ publication to IRB, NAMS.

Prof. Aarati Shah
Member Secretary, IRB, NAMS

Scanned with CamScanner
