# Supplementary material for: Intravenous Ringers lactate versus normal saline for predominantly mild acute pancreatitis in a Nepalese Tertiary Hospital
Source: PLoS One. 2022 Jan 28;17(1):e0263221. doi: 10.1371/journal.pone.0263221 (PMC9126573; doi:10.1371/journal.pone.0263221)
Supplement: S2 File — (PDF) [file pone.0263221.s003.pdf]

National Academy of Medical Sciences

Bir Hospital, Mahaboudha

Kathmandu Nepal

Proposal submitted for thesis in partial fulfillment of the requirement for the degree of DM (Gastroenterology)

Choice Of Initial Resuscitating Fluids In The Outcome Of Acute Pancreatitis

…………………

Dr. Binod Karki (MD, Internal Medicine)

Admission year: 2016

…………………………

Guide

Prof Dr Ramila Shrestha

……………………….

Member Secretary

IRB

CONTENTS

- Title
- Abbreviation
- Introduction
- Literature review
- Objectives
- Methodology
- Data collection
- Data analysis and statistical analysis
- Ethical consideration
- Time schedule
- Budget
- Performa
- Reference

ABBREVIATIONS:

AP: Acute Pancreatitis

CRP: C-Reactive Protein

RL: Ringer's Lactate

NS: Normal Saline

OF: Organ failure

SIRS: Systemic Inflammation response syndrome

ml : Milliliters

ER: Emergency

WBC: While blood cells

INTRODUCTION

Acute pancreatitis(AP) is a common diagnosis in gastroenterology department in any hospital presenting commonly as acute pain abdomen as shown in study of United states where AP was the most common reason for hospitalization (274,119 discharges)[1]. Early aggressive fluid resuscitation is recommended for initial management of acute pancreatitis.[2,3,4] As the inflammatory process progresses early in the course of the disease, there is an extravasation of protein-rich intravascular fluid into the peritoneal cavity and retroperitoneum, resulting in hemoconcentration and decreased renal perfusion with the associated elevation in BUN. Subsequently, the decreased perfusion pressure into the pancreas leads to microcirculatory changes that result in pancreatic necrosis.[5]. Fluid resuscitation is believed to play an important role in the prevention of complications such as pancreatic necrosis and organ failure by preserving pancreatic microcirculation.[6,7,8] However, recommendations for actual parameters for fluid replacement have been based solely on subjective expert opinion and, vigorous fluid resuscitation can be associated with potentially serious complications such as pulmonary sequestration.

Volume resuscitation with normal saline (NS) is standard treatment for the early phase of acute pancreatitis. However, infusion of large volumes of NS can lead to development of a hyperchloremic metabolic acidosis.[9,10,11] Recent data from experimental animal models indicate that acidosis is an important factor contributing to pathologic zymogen activation and severity of pancreatitis.[12,13]

The severity of inflammation in acute pancreatitis can be measured using Systemic Inflammation Response Syndrome (SIRS) score. SIRS is defined when two or more of the criteria are met viz Temperature<36 °C (96.8 °F) or >38 °C (100.4 °F), Heart rate>90/min, Respiratory rate>20/min and WBC (<4000/mm^3) or (>12,000/mm^3) or 10% bands with or without evidence of infection.[14] The score given to each of the parameter is one and ranges from zero i.e. no SIRS to four when all criteria are met. The severity of acute pancreatitis is greater among patients who have evidence of SIRS on day one and, in particular, among those with 3 or 4 SIRS criteria, compared with those without.[15]

C-reactive protein (CRP) is another marker of inflammation, which is an annular (ring-shaped), pentameric protein found in blood plasma, whose levels rise in response to inflammation. It is an acute-phase protein of hepatic origin that increases following interleukin-6 secretion by macrophages and T cells. Its physiological role is to bind to lysophosphatidylcholine expressed on the surface of dead or dying cells (and some types of bacteria) in order to activate the complement system via the C1Q complex.[16] In case of acute pancreatitis, CRP value correlates with the level of inflammation and the role of CRP in predicting the outcome in terms of local complications in acute pancreatitis has been shown to be equal with other bedside score.[17]

Though the early use of goal directed fluid therapy in the resuscitation of acute pancreatitis has been suggested by the guideline, it makes no recommendation whether normal saline or Ringer's lactate is to be used.[18] Choice of fluids amongst different crystalloids so far is based upon expert opinion only. In the present study, I will try to find out whether a more pH-balanced solution such as Ringer's Lactate (RL) could lead to a better outcome by reducing systemic inflammation compared with resuscitation with saline. The systemic inflammation will be measured using SIRS score and CRP both at baseline and during the treatment course.

LITERATURE REVIEW

Acute pancreatitis is an inflammatory condition of the pancreas characterized clinically by abdominal pain and elevated levels of pancreatic enzymes in the blood.[19] In a study done in United states, the incidence of AP in emergency department visits increased from 9.9 to 10.6 per 10,000 person-years over the period of 2006 to 2009 and represented a significant burden on US health care.[20] Gall stone followed by ethanol is the leading cause of AP worldwide.[5] As the inflammatory process progresses early in the course of the disease, there is an extravasation of protein-rich intravascular fluid into the peritoneal cavity and retroperitoneum, resulting in hemoconcentration and decreased renal perfusion with the associated elevation in BUN. Subsequently, the decreased perfusion pressure into the pancreas leads to microcirculatory changes that result in pancreatic necrosis.

Takeda K Mikami et al[21] evaluated the relationship between the angiographic abnormalities and the perfusion status of the pancreas documented by contrast-enhanced computed tomography (CE-CT) in 102 patients with ANP who were admitted during the early phase of the disease. Ischemic change with vasospasm on angiography of the intrapancreatic and extrapancreatic arteries was observed and corresponded with the poorly perfused area of the pancreas detected by CE-CT done at admission. Resultant pancreatic necrosis was confirmed on follow-up CE-CT examination consistent with the location where angiography demonstrated ischemic change with vasospasm. Hence, it was concluded that vasospasm in early phase of AP is associated with ischemia and subsequent necrosis, which can be minimized using aggressive fluid resuscitation. Hence, early aggressive fluid resuscitation is advocated to minimize hypotension and prevent these complications.

The advantage of early aggressive fluid therapy in AP is further highlighted by Wall I et al[22] in his study where series of patients with acute pancreatitis from a single community hospital in 1998 were compared to a consecutive series of patients with acute pancreatitis from the same institution in 2008. It was seen that significantly more patients developed pancreatic necrosis; 26 (15%) of 173 patients in 1998 compared to 4 (4%) of 113patients in 2008. The mean rate

of hydration was significantly higher in 2008 compared with that in 1998 (P = 0.02). In 1998, hydration was provided at 184 mL/h during the first 6 hours and 188 mL/h during the first 12 hours compared with 284 mL/h during the first 6 hours and 221 mL/h during the first 12 hours in 2008. There was a significant decrease in mortality in 2008 compared with that in 1998 (3.5% vs 12%, P = 0.03).

Some studies however argued against the routine use of early aggressive hydration. De-Madaria E et al[23] in their prospective cohort study, included consecutive adult patients with AP. Local complications and organ failure (OF) were defined according to the Atlanta Classification. Persistent OF was defined as OF of >48-h duration. Patients were divided into three groups according to the amount of fluid administered during the initial 24 h: group A: <3.1 liters, group B: 3.1-4.1 liters, and group C: >4.1 liters. A total of 247 patients were analyzed and it was seen than administration of >4.1 liters during the initial 24 h was significantly and independently associated with persistent OF, acute collections, respiratory insufficiency, and renal insufficiency. On the contrary, administration of <3.1 liters during the initial 24 h was not associated with OF, local complications, or mortality. Patients who received between 3.1 and 4.1 liters during the initial 24 h had an excellent outcome. Although this study raised concerns about the continuous use of aggressive hydration over 48 h, the role of early hydration (within the first 6 – 12 h) was not addressed. In addition, this study included sicker patients who would have required large volumes of hydration by the 48 h time point.

However the choice of fluid is still an area of research and the recommendations are based on expert opinion only. Several studies have been conducted with results favoring one crystalloid over another. Gardner TB[24] et al published a study in 2008 showing the lot of dilemma prevalent n viz amount of optimal resuscitative fluid, role for colloid solutions, clinical markers and the best time to start such fluids. This review described the microcirculation of the pancreas and the pathophysiologic alterations caused by acute pancreatitis. Previous animal experiments were described, as were the limited human studies specifically addressing fluid resuscitation. They also highlighted the need of investigations in existing recommendations and goals. In this review they tried to stimulate interest in this often overlooked subject.

Wu Bu[25] published randomized control trial showing the advantage of Ringer's Lactate in reducing systemic inflammation in AP. They included 40 patients with acute pancreatitis at three New England hospitals from May 2009-February 2010. Patients received goal-directed fluid resuscitation with RL solution, goal-directed fluid resuscitation with NS, standard fluid resuscitation with RL solution, or standard fluid resuscitation with NS. Systemic inflammation was measured on the basis of levels of systemic inflammatory response syndrome (SIRS) and C-reactive protein (CRP) level after 24 hours. It was shown that the volumes of fluid administered during a 24-hour period were similar among patients given goal-directed or standard fluid resuscitation (mean, 4300 vs 4600 mL, respectively; P = .87). Goal-directed resuscitation did not significantly reduce incidence of SIRS, compared with standard resuscitation (11.8% vs 13.0%, respectively; P = .85) or levels of CRP after 24 hours (87.1 vs 69.2 mg/dL, respectively; P = .75). By contrast, there was a significant reduction in SIRS after 24 hours among subjects resuscitated with RL solution, compared with normal saline (84% reduction vs 0%, respectively; P = .035); administration of RL solution also reduced levels of CRP, compared with NS (51.5 vs 104 mg/dL, respectively; P = .02). Hence they concluded that Patients with acute pancreatitis who were resuscitated with lactated Ringer's solution had reduced systemic inflammation compared with those who received saline.

Another triple blind, randomized control trial was conducted by Enrique de-Madaria et al[26] where fluid resuscitation was done with RL vs NS in AP. Patients admitted with AP were eligible. Patients were randomized to receive LR or NS. Primary outcome variables were number of systemic inflammatory response syndrome (SIRS) criteria at 24 hours, 48 hours and 72 hours and blood C-reactive protein (CRP) levels at 48 hours and 72 hours. In vitro complementary experiments were performed to further explore the interaction between pH, lactate and inflammation. Nineteen patients receiving LR and 21 receiving NS were analyzed. The median (p25–p75) number of SIRS criteria at 48 hours were 1 (1–2) for NS vs 1 (0–1) for LR, p=0.060. CRP levels (mg/l) were as follows: at 48 hours NS 166 (78–281) vs LR 28 (3–124), p=0.037; at 72 hours NS 217 (59–323) vs LR 25 (3–169), p=0.043. In vitro, LR inhibited the induction of inflammatory phenotype of macrophages and NF-kB activation. This effect was not observed when using Ringer's solution without lactate, suggesting a direct anti- inflammatory effect of lactate.

OBJECTIVES

Primary objective

To evaluate the effect of initial resuscitation with Ringers Lactate versus Normal Saline on the outcome of acute pancreatitis

Secondary objective

1. To analyze the difference in CRP in two groups as marker of systemic inflammation.

2. To analyze the difference in SIRS in two groups as marker of systemic inflammation.

3. To analyze the incidence of severity of diseases in both the groups as per the revised Atlanta classification.

4. To analyze the difference in complications of acute pancreatitis in both the groups.

5. To analyze the difference in length of hospital stay in both the groups

METHODOLOGY

Type of study: Open label randomized trial

Method of randomization: Envelop method for randomization

Place of Study: This study will be conducted in the Gastroenterology Unit of Bir Hospital.

Study Duration: Till the required sample size is met or one year whichever is more.

Inclusion criteria

All patients diagnosed with acute pancreatitis presenting in emergency (ER) of Bir hospital

Exclusion criteria

1. Patients below 18 yrs
2. Patients with co-morbid illness like chronic heart failure, chronic kidney disease.
3. Patients presenting to hospital for more than 48 hrs of symptoms or any transferred case from other hospital who are already resuscitated.
4. Patients with chronic obstructive pulmonary disease with requirement for home oxygen

Sample size calculation

For sample calculation we will be using CRP level as the change in the systemic inflammatory status. In a prospective cohort study [27] by Acevedo-Piedra NG et al , patients who received fluid resuscitation based on NS, mean blood CRP levels was 160 mg/l (standard deviation 111). We aim to detect a 100-mg/l difference with at least 80% power, at a two-sided alpha level of 5%.

Using the following formula for estimating sample size:

$$\mathbf{n1=(\sigma 1^2+\sigma 2^2/K)(z1-\alpha/2+z1-\beta)^2/\Delta^2}$$

It is calculated that 25 minimum patients in each arm would be required to have 95% confidence interval and power of 80% however all the patients even beyond the sample size during the study period of one year will be included.

Intervention details:

All the patients who presented to the ER of Bir Hospital and diagnosed as acute pancreatitis using the Atlanta classification will be subjected to fluid resuscitation using either RL or NS 10 ml/kg of the study fluid in 60 minutes immediately after randomization, and then 1.5 ml/kg/hour of the study fluid for three days. All patients will receive additional 1 liter of 5% dextrose per 24 hours. The amount of daily fluid administered were as per the standard guideline which is 250-400 ml per hour.[5] Additional potassium chloride is added as per daily serum biochemistry report. Adequate fluid replacement will be assessed by an improvement in vital signs (goal heart rate <120 beats/minute, mean arterial pressure between 65 to 85 mmHg), urine output (>0.5 to 1 cc/kg/hour) and reduction in hematocrit (goal 35 to 44 percent) and BUN over 24 hours, particularly if they were high at the onset. All patients will be followed till the time of hospital discharge.

Case definition

Case definition of acute pancreatitis and its severity will be done as per the revised Atlanta Classification.[28]

Diagnosis of Acute Pancreatitis: The diagnosis of acute pancreatitis requires two of the following three features: (1) abdominal pain consistent with acute pancreatitis (acute onset of a persistent, severe, epigastric pain often radiating to the back); (2) serum lipase activity (or amylase activity) at least three times greater than the upper limit of normal; and (3) characteristic findings of acute pancreatitis on contrast-enhanced computed tomography (CECT) and less commonly magnetic resonance imaging (MRI) or transabdominal ultrasonography.

Severity of Acute Pancreatitis Acute pancreatitis is graded as mild, moderately severe and severe as follows.

Mild acute pancreatitis: No organ failure, No local or systemic complications,

Moderately severe acute pancreatitis: Organ failure that resolves within 48 h (transient organ failure) and/or local or systemic complications without persistent organ failure

Severe acute pancreatitis: Persistent organ failure (>48 h) either Single or multiple organ failure.

The SIRS score and CRP titre of all the patients will be recorded at admission and will be repeated again at 72 hours during hospital stay. SIRS score will be recorded as not present or if present as score 1-4. Each of the criteria shown below will be given one score.[14]

- Temperature<36 °C (96.8 °F) or >38 °C (100.4 °F)
- Heart rate>90/min
- Respiratory rate>20/min
- WBC(<4000/mm^3) or (>12,000/mm^3) or 10% bands

CRP measurement will be done by turbidometry method and expressed in quantitative CRP as mg/dl.

Statistical analysis

All data will be analyzed on an intent-to-treat basis. Differences between the two groups with continuous data will be assessed using student-t test for normal and Mann-Whitney U test for non-normal distributions. Logistic regression will be used to calculate the significance of difference if found between two groups in terms of outcome. A two-sided p value of less than 0.05 was considered statistically significant. All statistical calculations were performed with SPSS

DUMMY TABLES

1. Baseline characteristics in two groups

	**Normal saline**	**Ringer's Lactate**	**p Value**
Age			
Gender			
Etiology			
SIRS on admission			
CRP on admission			
Urea			
Creatinine			
WBC			
Hb			
Amylase			

Table 2. Systemic inflammatory response syndrome (SIRS)criteria according to study fluid.

**Time from randomization**	**Variable**	**Normal Saline**	**Ringer's actate**	**p Value**
Basal	No of SIRS criteria (1-4)			
24 hrs				
72 hrs				

Table 3. Quantitative CRP measurement according to study fluid

**Time from randomization**	**Normal Saline**	**Ringer's Lactate**	**p Value**
Basal – 24 hrs			
72 hrs			

Table 4. The distribution of severity of acute pancreatitis between two groups

	**Total**	**Normal Saline**	**Ringer's Lactate**	**p Value**
Mild AP				
Moderate AP				
Severe AP				

Table 5. Hospital length stays between two groups

	**Normal Saline**	**Ringer's Lactate**	**p Value**
Hospital stay in days (mean)			

Table 6. Complications of pancreatitis (local) in both the groups

Complications (local)	**Normal Saline**	**Ringer's Lactate**	**p Value**
1. Peripancreatic fluid collections			
2. Acute necrotic collection			
3. Pseudocyst			
4. Walled off necrosis			

ETHICAL CONSIDERATION

The study will be started after the approval obtained from Institutional Review Board (IRB) of National Academy of Medical Sciences and a written informed consent will be obtained from all patients included in this study.

Patients will be assured of full confidentiality during and after the study period.

Written consent form will be developed and be given to patient/patient attendant. The consent will be taken only after the better understanding of the advantages, disadvantages and complications of the procedures.

INFORMED CONSENT

1. Participants can withdraw from the study at any time without giving any reason at anytime during study period.

2. Statement guaranteeing confidentiality of the research participant.

3. A statement indicates that the participant has understood all information in the consent form and is willing to participate in the research. Participant will be explained about the diseases, the study, procedures and its significance, the benefits that they can expect as well as the benefit that it is going to provide to the community in the language he /she understands before obtaining the consent, their queries will be answered. If possible, a witness is present during the procedure. Patient will not be discriminated from the treatment failing to participate in the study.

4. The principal investigator will be responsible for obtaining informed consent from the participants in the study. There will be nothing withheld from the participants at time when informed consent is being sought.

LETTER OF CONSENT

I ………am willingly participating in the study titled "**Choice of initial resuscitating fluids in the outcome of acute pancreatitis**'', being conducted in NAMS, bir hospital. I have been fully explained about the details of the study. I have been assured that confidentiality will be maintained to the up most. I am also aware that I have full rights to withdraw my participation from the study whenever I wish to do so .My treatment will not be compromised even if I drew myself from the study .I will neither be charged extra costs nor be paid any extra benefits regarding this study. .

………………………….	----------------------
Signature of participant	Consent taken by
Name:	Name:
Address:	Place:
Contact No.-	
Date:	

मन्जू ु रीनामा

म ....मे रो स्वे च्छाले नै यस चि. वि. रा. प्र ., मा ँ **Choice of initial resuscitating fluids in the outcome of acute pancreatitis"** सम्बन्धी अध्ययन–अनु सन्धानमा सहभागी हु न राजी छु । मलाई यस अनु सन्धानको बिधि, फाइदा ,असर र गो पनियता साथै भविष्यमा यसको प्र यो जनको बारे मा सम्पु र्ण जानकारी दिइएको छ र मलाई यसमा कु नै आपत्ती छै न । साथै यस अनु सन्धानमा सहभागी भए बापत मलाई कु नै शु ल्क लाग्ने छै न र थप सु विधा पनि दिइने छै न । परिक्षणमा लाग्ने खर्च अनु सन्धानकर्ता आफै ले बे हो रिने छ । कु नै पनि बखत मै ले यस अनु सन्धानमा सहभागी हु न नचाहे को खण्डमा पनि मे रो उपचारमा कु नै बाधा पारिने छै न।

औ ं ठाछाप

दायाँ

बाया

सहभागी

नाम :

हस्ताक्षर :

ठे गाना:

मितिः

TIME SCHEDULE

Data collection will be done in a period of 1 year.

During this period literature review will be undertaken from relevant journals, internet websites and standard text books. Other information and experiences will be discussed with colleagues, seniors, guide, co-guide and faculties.

Data will be stored and analyzed as mentioned. The data collection will start from getting permission of IRB and will continue until samples more than 40 patients meeting the inclusion criteria and excluded by the exclusion criteria are obtained.

The data will be analyzed after that over two weeks and the final thesis write up will be carried out over 4 to 6 weeks and thesis will be submitted for its correction within 4-6 month.

The final thesis is planned to be submitted on first half 2019.

GANTT CHART

2017 **2018** **2019**

Date Activates	S	O	N	D	J	F	M	A	M	J	J	A	S	O	N	D	J	F
Literature review																		
Topic selection																		
Ground work																		
Proposal writing																		
Proposal submitting																		
Data collection																		
Data analysis																		
Thesis writing																		
Submittin g thesis																		
Final thesis writing																		
Final thesis submitting																		

REFERENCES

[1] Peery AF, Dellon ES, Lund J, et al. Burden of gastrointestinal disease in the United States: 2012 update. Gastroenterology 2012; 143:1179

[2] Working party of the British Society of Gastroenterology UK guidelinesfor the management of acute pancreatitis. Gut 2005;54(Suppl 3):iii1–iii9

[3] Tenner S, Baillie J, DeWitt J,Vege SS.American College of Gastroenterology Guideline: Management of Acute Pancreatitis.Am J Gastroenterol.2013.

[4] Sarr MG. IAP guidelines in acute pancreatitis. Dig Surg 2003;20:1–3.

[5]Tenner S, Steinberg WM.Acute Pancreatitis.In:Mark Feldman, Lawrence S. Friedman, Lawrence J. Brandt.Sleisenger and Fordtran's Gastrointestinal and Liver Disease.10th ed.USA.Elsevier;2016.p. 969-93

[6] Knoefel WT, Kollias N, Warshaw AL.Pancreatic microcirculatorychanges in experimental ancreatitis of graded severity in the rat. Surgery.1994;116:904–13.

[7]Pandol SJ, Saluja AK, Imrie CW. Acute pancreatitis: Bench to the bedside. Gastroenterology.2007;133:e1–e25.

[8] Strate T, Mann O, Kleinhans H. Microcirculatory functionand tissue damage is improved after therapeutic injection of bovine hemoglobin in severe acute rodent pancreatitis. Pancreas.2005;30:254–259.

[9] Scheingraber S, Rehm M, Sehmisch C. Rapid saline infusionproduces hyperchloremic acidosis in patients undergoing gynecologic surgery. Anesthesiology 1999;90:1265–1270.

[10] Reid F, Lobo DN, Williams RN.(Ab)normal saline andphysiological Hartmann's solution: a randomized double-blind crossover study. Clin Sci (Lond).2003;104:17–24.

[11] Wilkes NJ, Woolf R, Mutch M. The effects of balancedversus saline-based hetastarch and crystalloid solutions on acid-base and electrolyte status and gastric mucosal perfusionin elderly surgical patients. Anesth Analg 2001;93:811–16

[12] Noble MD, Romac J, Vigna SR. A pH-sensitive, neurogenicpathway mediates disease severity in a model of post-ERCP pancreatitis. Gut 2008;57:1566–71.

[13] Bhoomagoud M, Jung T, Atladottir J. Reducing extracellularpH sensitizes the acinar cell to secretagogue-induced pancreatitisresponses in rats. Gastroenterology 2009;137:1083

[14] American College of Chest Physicians/Society of Critical Care Medicine Consensus Conference: Definitions for sepsis and organ failure and guidelines for the use of innovative therapies in sepsis. Crit Care Med. 1992 Jun;20(6):864-74.

[15] Singh VK, Wu B, Bollen TI, Repas K,Maurer R,Mortele KJ et al.Early systemic inflammatory response syndrome is associated with severe acute pancreatitis.Clinical Gastroenterology and Hepatology 2009;7:1247–1251.

[16] Thompson D, Pepys MB, Wood SP.The physiological structure of human C-reactive protein and its complex with phosphocholine.Structure.1999;7(2):169–77.

[17] Vinish DB,Abishek V,Sujatha K,Arulprakash S,Solomon R5, Ganesh P.Role of bedside pancreatic scores and C-reactive protein in predicting pancreatic fluid collections and necrosis.Indian J Gastroenterol. 2017 Jan;36(1):43-49.

[18] Crockett SD,Wani S,Gardner TB,Falck-Ytter Y,Barkun A.American Gastroenterological Association Institute Guideline on Initial Management of Acute Pancreatitis.Gastroenterology 2018;-:1–6

[19] Sarles H. Revised classification of pancreatitis--Marseille 1984. Dig Dis Sci 1985; 30:573.

[20] McNabb-Baltar J, Ravi P, Isabwe GA, Suleiman SL, Yaghoobi M, Trinh QD et al. A population-based assessment of the burden of acute pancreatitis in the United States. Pancreas.2014 Jul;43(5):687-91.

[21] Takeda K1, Mikami Y, Fukuyama S, Egawa S, Sunamura M, Ishibashi T et al. Pancreatic ischemia associated with vasospasm in the early phase of human acute necrotizing pancreatitis.Pancreas. 2005 Jan;30(1):40-9.

[22] Wall I,Badalov N,Baradarian R. Decreased morbidity and mortality in patients with acute pancreatitis related to aggressive intravenous hydration.Pancreas.2011;40:547–50.

[23] De-Madaria E,Soler-Sala G,S á nchez-Paya J.Infl uence of fl uid therapy on the prognosis of acute pancreatitis: a prospective cohort study.Am J Gastroenterol 2011;106:1843–50.

[24] Gardner TB, Vege SS, Pearson RK, Chari ST. Fluid resuscitation in acute pancreatitis. Clin Gastroenterol Hepatol 2008; 6:1070.

[25] Wu BU,Hwang JQ,Gardner TH.Lactated Ringer's solution reduces systemic inflammation compared with saline in patients with acute

pancreatitis.Clin Gastroenterol Hepatol.2011;9:710–17 .

[26] Enrique de-Madaria1,Iva´n Herrera-Marante,Vero´nica Gonza´lez-Camacho,Laia Bonjoch, Noe´ Quesada-Va´zquez,Isabel Almenta-Saavedra et al.Fluid resuscitation with lactated Ringer's solution vs normal saline in acute pancreatitis:A triple-blind, randomized, controlled trial.United European Gastroenterology Journal.2017.

[27] Acevedo-Piedra NG, Moya-Hoyo N, Rey-Riveiro M. Validation of the determinant-based classification and revision of the Atlanta classification systems foracute pancreatitis. Clin Gastroenterol Hepatol 2014; 12:311–16.

[28] Banks PA,Bollen TL,Dervenis C,Gooszen HG,Johnson CD,Sarr MG et al.Classification of acute pancreatitis—2012: revision of the Atlanta classification and definitions by international consensus.Gut 2013;62:102–111
